# Evaluating In-Hospital Arrhythmias in Critically Ill Acute Kidney Injury Patients: Predictive Models, Mortality Risks, and the Efficacy of Antiarrhythmic Drugs

**DOI:** 10.3390/jcm14134552

**Published:** 2025-06-26

**Authors:** Wanqiu Xie, Henriette Franz, Toma Antonov Yakulov

**Affiliations:** 1 Renal Division, Department of Medicine, Faculty of Medicine and Medical Center-University of Freiburg, 79106 Freiburg, Germany; wanqiu.xie@uniklinik-freiburg.de; 2 Department of Biomedicine, University of Basel, Pestalozzistr. 20, CH-4056 Basel, Switzerland; henriette.franz@unibas.ch

**Keywords:** acute kidney injury, arrhythmias, XGBoost, Bayesian information criterion, in-hospital mortality, antiarrhythmic medications

## Abstract

**Background:** Acute kidney injury (AKI) in critically ill patients is often complicated by arrhythmias, potentially affecting outcomes. This study aimed to develop predictive models for arrhythmias in AKI patients and assess the impact of antiarrhythmic drugs on in-hospital mortality. **Methods:** We conducted a multi-database retrospective cohort study using MIMIC-IV and eICU databases. XGBoost and Bayesian Information Criterion (BIC) models were employed to identify key predictors of arrhythmias. Weighted log-rank and Cox analysis evaluated the effect of amiodarone and metoprolol on in-hospital mortality. **Results:** Among 14,035 critically ill AKI patients, 5614 individuals (40%) developed arrhythmias. Both XGBoost and BIC showed predictive power for arrhythmias. The XGBoost model identified HR_max, HR_min, and heart failure as the most important features, while the BIC model highlighted heart failure had the highest odds ratio (OR 1.18, 95% CI 1.16–1.20) as a significant predictor. Patients experiencing arrhythmia is associated with in-hospital mortality (arrhythmia group: 636 (11.3%) vs. non-arrhythmia group: 587 (7.0%), *p* < 0.01). Antiarrhythmic medications showed a statistically significant effect on in-hospital mortality (amiodarone: HR 0.28, 95% CI 0.19–0.41, *p* < 0.01). **Conclusions:** Our predictive models demonstrated a robust discriminatory ability for identifying arrhythmia occurrence in critically ill AKI patients, with identified risk factors showing strong clinical relevance. The significant association between arrhythmia occurrence and increased in-hospital mortality underscores the clinical importance of early identification and management. Furthermore, amiodarone therapy effectively reduced the risk of in-hospital mortality in these patients, even after accounting for time-dependent biases. The findings highlight the necessity of precise arrhythmia definition, careful consideration of time-dependent covariates, and comprehensive model validation for clinically actionable insights.

## 1. Introduction

Acute kidney injury (AKI) is a common and life-threatening condition in intensive care unit (ICU) patients, associated with a significant increase in mortality rates [1]. Characterized by an abrupt decline in renal function, typically evidenced by a rapid rise in serum creatinine levels and a reduction in urine output, AKI often leads to a cascade of systemic disturbances. These disturbances can profoundly impact cardiovascular stability, predisposing patients to various cardiac arrhythmias [2,3]. The intricate relationship between AKI and cardiac arrhythmias is multifaceted, with pathophysiological mechanisms highlighting electrolyte imbalances such as hyperkalemia and hypocalcemia as critical risk factors for arrhythmic events. Furthermore, other metabolic derangements frequently observed in AKI, including hyper- or hypophosphatemia, hypomagnesemia, and metabolic acidosis, can exacerbate myocardial irritability and trigger a spectrum of arrhythmias [4].

Despite the recognized association between AKI and cardiovascular complications, the specific incidence and characteristics of arrhythmias in critically ill AKI patients remain an area requiring more comprehensive investigation. While broader studies have reported arrhythmia incidence in general ICU populations ranging from 12% to 78% [5], data specifically focusing on AKI patients are less abundant. New-onset atrial fibrillation (NOAF), for instance, is a common arrhythmia in critically ill patients, with reported incidences ranging from 14% to 37% in AKI patients [6,7]. This underscores the clinical significance of understanding and addressing arrhythmias in this vulnerable patient group, as their occurrence can further compound the already high mortality risk associated with AKI.

Previous research has extensively explored various AKI-related complications, such as liver failure and pulmonary edema [8,9,10,11,12]. However, a dedicated focus on the epidemiology, underlying causes, and prognostic implications of arrhythmias in critically ill AKI patients has been notably limited. This knowledge gap impedes the development of targeted diagnostic and therapeutic strategies. Therefore, this study aims to bridge this gap by comprehensively exploring the underlying causes and predictive factors of arrhythmias in AKI patients. We use advanced analytical methods, including Extreme Gradient Boosting (XGBoost) and Bayesian Information Criterion (BIC) analysis, to identify key determinants of arrhythmia occurrence. Furthermore, another objective of this research is to examine the impact of arrhythmias on in-hospital mortality in this patient cohort. Finally, we assess the efficacy of antiarrhythmic medications in mitigating the risk of in-hospital death, providing insights into potential therapeutic interventions to improve outcomes for critically ill AKI patients.

## 2. Methods

### 2.1. Data Sources

The primary dataset for this study was obtained from the Medical Information Mart for Intensive Care IV (MIMIC-IV, a publicly accessible critical care database managed by the Massachusetts Institute of Technology (MIT) and Beth Israel Deaconess Medical Center (BIDMC) [13]. MIMIC-IV compiles detailed patient data from critical care units at Beth Israel Deaconess Medical Center in Boston, Massachusetts. For external validation, we utilized the eICU Collaborative Research Database, which encompasses a diverse array of ICU data from over 200,000 admissions across multiple hospitals in the United States [14]. Notably, the hospitals contributing data to MIMIC-IV did not participate in the eICU program, ensuring an independent validation set. Both databases were used in compliance with the approvals granted by the Institutional Review Boards of MIT and BIDMC. The requirement for informed consent was waived for this study, under certification number 46025826 for author Wanqiu Xie.

### 2.2. Participant Selection and Inclusion Criteria

The inclusion criteria for participants in this study were defined as follows:

1. As our objective was to identify arrhythmias occurring in patients with acute kidney injury (AKI), we first identified AKI cases using the KDIGO criteria [12]. To enhance clinical validity, we further verified these cases by confirming a corresponding ICD-10 diagnostic code for AKI. In total, we included 6,217,152 creatinine-based abnormal records from the MIMIC-IV database and 101,261 records from the eICU database (note that a single patient may have multiple laboratory entries over time).
2. Arrhythmias were defined based on diagnostic reports generated after the patient underwent an electrocardiogram (ECG) examination. The arrhythmia types analyzed included atrial fibrillation (AF), sinus tachycardia (ST), ventricular tachycardia (VT), sinus bradycardia, first-degree atrioventricular block (1st AV block), right bundle branch block (RBBB), left bundle branch block (LBBB), second-degree AV block (2nd AV block), third-degree AV block (3rd AV block), atrial flutter, and supraventricular tachycardia (SVT).
3. We excluded patients who were ≤18 years old.
4. Only the first ICU admission for each patient was retained; subsequent admissions were excluded.
5. Patients with a documented arrhythmia prior to the diagnosis of AKI were excluded to ensure temporal causality.

**Figure 1:**
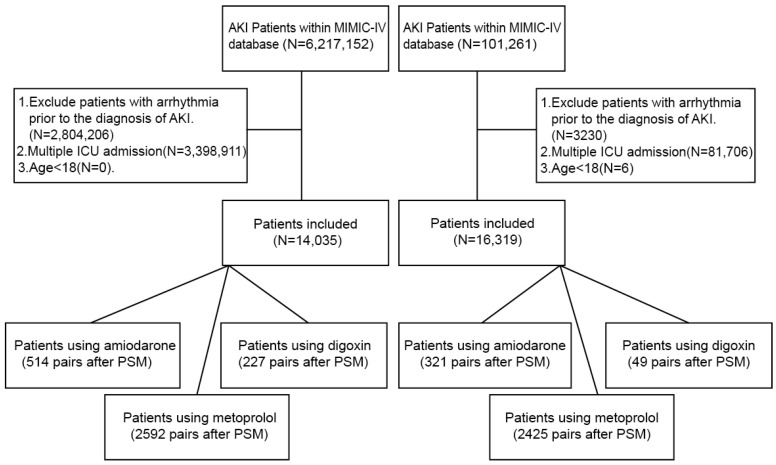
Flowchart of patient selection.

The detailed flowchart of the patient selection process is presented in Figure 1. In addition, the eICU Collaborative Research Database was used as an independent external test set to evaluate the generalizability and applicability of the predictive models.

After applying these criteria, the final sample included 14,035 patients from the MIMIC-IV database and 16,319 patients from the eICU Collaborative Research Database. The screening process for participant selection is illustrated in Figure 1. Additionally, models derived from the eICU Collaborative Research Database served as an independent testing set to evaluate the generalizability and applicability of the established predictive models.

### 2.3. Variable Selection

For this study, a comprehensive set of variables was extracted from the MIMIC-IV and eICU databases at the time of ICU admission. These variables were categorized into several groups: (a) demographic information: age, gender, length of stay (LOS), in-hospital mortality; (b) comorbidities: cerebral infarction, chronic kidney disease (CKD), diabetes, heart failure, hypertension, infarct circulation, pancreatitis, arrhythmia; (c) medication usage: insulin, antibiotic usage, blood products, crystalloids, furosemide, nitroglycerin, colloids, anticoagulant, pressor, sodium bicarbonate (SB) use; (d) laboratory results: the initial values, the lowest and highest levels within the first 24 h after the ICU admission of following variables: white blood cells (WBC), anion gap (AG), blood bicarbonate, blood urea nitrogen (BUN), blood chloride, serum creatinine, blood glucose, hemoglobin, international normalized ratio (INR), blood potassium, blood sodium, blood pH, platelet (PLT), prothrombin time (PT), red blood cells (RBC), total calcium; (e) vital signs: the initial values, the lowest and highest levels within the first 24 h after the ICU admission of following variables: acute physiology score III (APSIII), mean arterial pressure (MAP), heart rate (HR), respiratory rate (RR), temperature, and the urine output (every hour record); and (f) ICU treatment measures: ventilation, renal replacement therapy (RRT).

Variables with more than 30% missing data, such as alkaline phosphatase, and free blood calcium, were excluded from the analysis. The proportion of missing data for each variable is provided in Supplementary Table S1. The missing values for the included variables were imputed using multiple imputation by chained equations (MICE) prior to data analysis [13,14]. Specifically, we employed the random forest method (method = “rf”) within the MICE framework, with 5 imputations (m = 5) and 10 iterations (maxit = 10). To assess convergence, we evaluated autocorrelation (AC) and the potential scale reduction factor (PSRF), which are summarized in Supplementary Table S2. These diagnostics indicate acceptable convergence across imputations [15,16].

The primary outcome of our study was the risk of developing arrhythmias during hospitalization among patients with AKI, defined as the presence of abnormal ECG findings occurring after the patient’s serum creatinine first met the KDIGO criteria for AKI. Secondary outcomes included whether different types of arrhythmias among ICU patients with AKI were associated with an increased risk of in-hospital mortality and whether the use of antiarrhythmic drugs in ICU patients with AKI and arrhythmias was associated with improved in-hospital outcomes.

Definition and handling of antiarrhythmic drug exposure: For digoxin, given its narrow therapeutic window, rapid onset, and potential toxicity, we considered patients exposed if they received at least one intravenous dose during their ICU stay. For amiodarone and metoprolol, we defined exposure as receiving at least three consecutive intravenous doses, regardless of whether it was administered as three times within one day, once daily over three days, or twice daily for more than 1.5 days. This definition was established to ensure therapeutic dosing and minimize transient or single-dose exposure misclassification.

### 2.4. Statistical Analysis

The initial step involved testing the normality of baseline data using both the Agostino and Anscombe tests. Normally distributed variables were presented as mean ± standard deviation (SD) and analyzed using the *t*-test, while non-normally distributed variables were expressed as interquartile ranges (IQR) and assessed using the Wilcoxon rank-sum test or the chi-squared test [17].

To identify key variables within our dataset, we employed Bayesian Information Criterion (BIC) optimal subset regression and Extreme Gradient Boosting (XGBoost) [18,19,20,21].

All models underwent external validation. Additionally, the MIMIC-IV dataset was split into 70% for training and 30% for internal validation. The machine learning XGBoost model was appraised based on accuracy, precision, recall, and F1 score metrics, both internally and externally. Selected variables were incorporated into regression models, and their performance was evaluated using calibration plots. Decision Curve Analysis (DCA) was used to evaluate the clinical utility of the prediction model. Net benefit was calculated across a range of threshold probabilities by comparing the model to default strategies of treating all or none.

To investigate the association between antiarrhythmic drug exposure and in-hospital mortality among ICU patients with acute kidney injury (AKI), we followed a stepwise modeling strategy. First, we performed propensity score matching (PSM) to reduce confounding bias between the exposed and unexposed groups. Matching was conducted using a 1:1 nearest-neighbor algorithm, based on a comprehensive set of baseline covariates including demographics, comorbidities, and illness severity markers. Standardized mean differences (SMDs) were calculated to assess balance. Second, we conducted Kaplan–Meier (KM) survival analysis on the matched cohort. The ICU admission time was defined as the common time origin, enabling a static evaluation of survival differences under the assumption of fixed exposure status. Compared to the static evaluation in KM analysis, we subsequently conducted a dynamic analysis using time-varying covariate Cox models to more accurately capture treatment exposure over time. To address this, we subsequently constructed Cox proportional hazards models with time-varying covariates. For each antiarrhythmic drug (e.g., amiodarone), we generated a time-dependent variable (e.g., amiodaronetime) coded as “0” before drug initiation and “1” after. The model employed the counting process framework: Surv(start, stop, event), where start and stop corresponded to ICU time intervals relative to admission, and event denoted in-hospital death. This method allows for proper modeling of dynamic treatment exposure and avoids the biases inherent in static survival models. Covariates included in the model were selected based on clinical relevance. We used a time-varying covariate Cox model to address potential non-proportional hazards and dynamic drug exposure during ICU stay. Only when both KM and time-varying Cox models demonstrated consistent and statistically significant results did we proceed to validate these findings using Fine–Gray competing risk models [20,21].

Statistical analyses were conducted using R 4.1.1 (R Core Team, 2022). The R packages used in this study can be found in the Supplementary Materials. Statistical significance was established at a *p*-value threshold of less than 0.01.

## 3. Results

### 3.1. Patient Characteristics

Our analysis of the MIMIC-IV database identified 14,035 critically ill patients diagnosed with AKI. Among this study, 5614 individuals (40%) developed arrhythmias during their hospital stay. Table 1 presents the findings from our univariate analysis, comparing AKI patients who experienced arrhythmias to those who did not. Interestingly, we observed statistically significant difference in illness severity between the two groups upon ICU admission, as measured by the APSIII scores (arrhythmia group: 51 [40–67] vs. non-arrhythmia group: 47 [37–62], *p* < 0.01). In addition, we found that whether the patient experiences arrhythmia is associated with in-hospital mortality (arrhythmia group: 636 (11.3%) vs. non-arrhythmia group: 587 (7.0%), *p* < 0.01).

Our analysis revealed several significant differences between the groups. Patients who developed arrhythmias were generally older and had a higher prevalence of certain comorbidities, including CKD, hypertension and heart failure. Moreover, this group showed distinct patterns in medication use, with higher rates of antibiotic administration, furosemide prescription, and nitroglycerin use.

We also identified significant variations in several clinical parameters measured within the first 24 h of admission. These differences were observed in blood test result levels (BUN_valuenum, BUN_max, BUN_min, chloride_valuenum, chloride_max, chloride_min, creatinine_valuenum, creatinine_min, totalcalcium_valuenum, glucose_max), coagulation markers (INR_valuenum, INR_max, INR_min, PLT_valuenum, PLT_max, PT_valuenum, PT_max, PT_min), acid-base balance (pH_valuenum, pH_max, bicarbonate_valuenum, bicarbonate_max, bicarbonate_min, AG_valuenum, AG_min), and cardiovascular parameters (APSIII, MAP_valuenum, MAP_max, MAP_min, HR_valuenum, HR_max, HR_min, RR_valuenum RR_max, RR_min, temperature_valuenum, temperature_max, temperature_min, output_valuenum, output_max, output_min).

### 3.2. XGBoost

**Figure 2:**
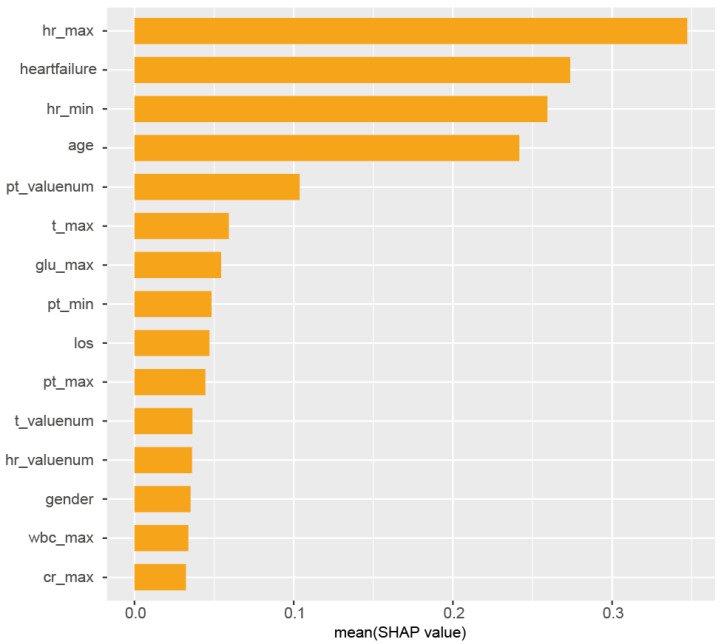
Feature importance derived from the XGBoost model. The x-axis represents the gain, which indicates the relative contribution of each feature to the model. Key features include hr_max, heartfailure, hr_min, age, and pt_valuenum. Abbreviations: hr = heart rate, map = mean arterial pressure, plt = platelet, glu = glucose, t = temperature, pt = prothrombin time, ckd = chronic kidney disease, wbc = white blood cell, cr = creatinine, los = length of stay. Suffixes: _valuenum indicates the initial value, _min represents the minimum value, and _max represents the maximum value obtained during the first 24 h in the ICU.

We employed the XGBoost machine learning algorithm to develop our predictive model. After data normalization, we trained the model using the following hyperparameters: nrounds = 75, max_depth = 3, eta = 0.1, gamma = 0.25, colsample_bytree = 1, min_child_weight = 1, and subsample = 0.25. The model-based importance of features was established, and the relative importance of predictors in the XGBoost model is illustrated in Figure 2. The model identified hr_max (maximum heart rate), comorbid heart failure, hr_min (minmum heart rate), age, and the initial PT value as the most important features for predicting arrhythmias in AKI patients. The influence of the top 15 risk factors on prediction outcomes, including their direction of impact, is detailed in Supplementary Figure S1. Supplementary Figure S2 presents the SHAP values, elucidating the contribution of individual features to the final prediction, enhancing model interpretability for individual patient outcomes.

### 3.3. BIC Best Subset Selection

**Figure 3:**
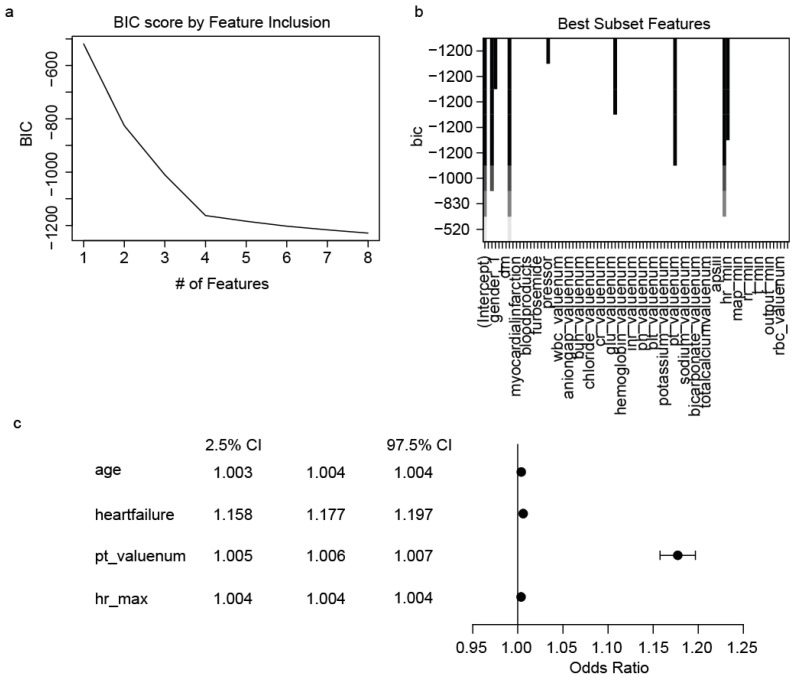
Feature selection using Bayesian Information Criterion (BIC) algorithm. (**a**) BIC score as a function of the number (#) of features included in the model. The BIC score decreases rapidly with the inclusion of the first few features, then continues to decline more gradually as additional features are added. (**b**) BIC scores for individual features. The x-axis lists the features, and the y-axis shows the BIC scores. Lower scores indicate better predictive power. The top features with the largest absolute value BIC scores include age, heart failure, pt_valuenum and hr_max, suggesting these are among the most important predictors in the model. (**c**) Forest plot showing the odds ratios (OR) and 95% confidence intervals (CI) for selected features in the BIC model. hr_max refers to the maximum heart rate calculated based on values obtained during the first 24 h in the ICU, and pt_valuenum refers to the prothrombin time initial value within 24 h of the patient’s admission to the ICU.

We applied the Bayesian Information Criterion (BIC) Best Subset Selection method to 88 candidate variables. This approach identified four key predictors of arrhythmia, with a variable-to-observation ratio of 22:1 (Figure 3a). These critical variables include heart failure, age, pt_valuenum and maximum heart rate. Figure 3b displays the BIC scores for these variables, indicating their relative importance in prediction; a higher absolute value of the score indicates greater predictive ability. The odds ratios (ORs) for these variables in the regression model are presented in Figure 3c. Age, pt_valuenum, hr_max, and heart failure were among the most influential predictors in the BIC model. Notably, heart failure had the highest odds ratio (OR 1.18, 95% CI 1.16–1.20), indicating a strong association with arrhythmia risk.

### 3.4. Subgroup Analysis and Models Performance

As part of the sensitivity analysis, we further constructed separate XGBoost models using individual arrhythmia subtypes (sinus bradycardia, atrial fibrillation, and sinus tachycardia) as outcome variables to explore potential outcome heterogeneity. Interestingly, these subgroup models exhibited a predictive performance compared to that of the overall model (Table 2). Despite differences in discrimination metrics, the top-ranked predictive variables remained consistent across subgroups, indicating that the model’s key features were robust and generalizable across different arrhythmia phenotypes (Supplementary Figures S3–S5).

The XGBoost model achieved an Area Under the Curve (AUC) of 0.671 in the internal validation set. When validated using patient data from the eICU database, the AUC decreased to 0.616. Table 2 summarizes the model’s performance metrics, including Accuracy, Precision, Recall, and F1 Score, for both internal and external validation. These results indicate that the XGBoost model has discriminative ability across both validation sets. The model performs moderately, with poor recall, but possesses discriminatory ability.

**Figure 4:**
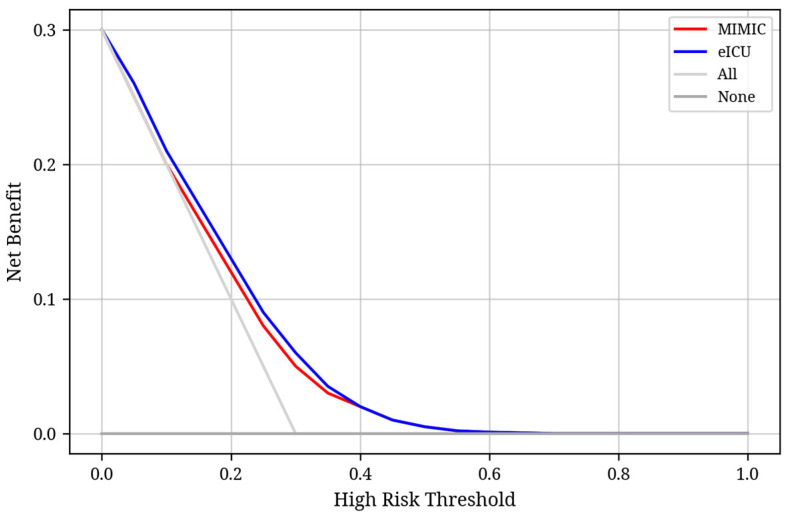
Decision curve analysis (DCA) for the prediction model in MIMIC and eICU cohorts. The red and blue lines represent the net benefit of the model in the MIMIC and eICU cohorts, respectively, across a range of high-risk thresholds. The gray and black lines correspond to the “treat all” and “treat none” strategies. Both models showed a higher net benefit than default strategies within a clinically relevant threshold range (approximately 0.05 to 0.35), supporting their potential utility for early clinical decision-making.

The regression model constructed using BIC-selected variables demonstrated a similar performance, with an AUC of 0.66 in the internal validation set and 0.68 in the external validation set. Supplementary Figure S6 illustrates the calibration curves for both the MIMIC-IV and the eICU validation set. Its well-calibrated predictions indicate that the estimated probabilities accurately reflect the event rates observed, supporting its potential value in risk stratification. We conducted decision curve analysis (DCA) to evaluate the clinical utility of the prediction model in both internal (MIMIC) and external (eICU) validation cohorts. As shown in Figure 4, the model demonstrated a consistently higher net benefit than the “treat all” and “treat none” strategies across a range of threshold probabilities, particularly between 0.05 and 0.35, which is typically considered clinically actionable. The overlapping curves in both cohorts suggest that the model’s decision-making value generalizes well across different populations.

### 3.5. Prognostic Factor Analysis

To ensure a balanced comparison, we employed 1:1 propensity score matching (PSM) for each arrhythmia group before examining the relationship between arrhythmias and in-hospital mortality. Subgroup analyses, presented in Table 3, demonstrated that both atrial fibrillation and sinus tachycardia were associated with higher mortality rates (the PSM-matched data for the subgroup analysis are shown in Supplementary Tables S3–S7).

### 3.6. Intervention Analysis

**Figure 5:**
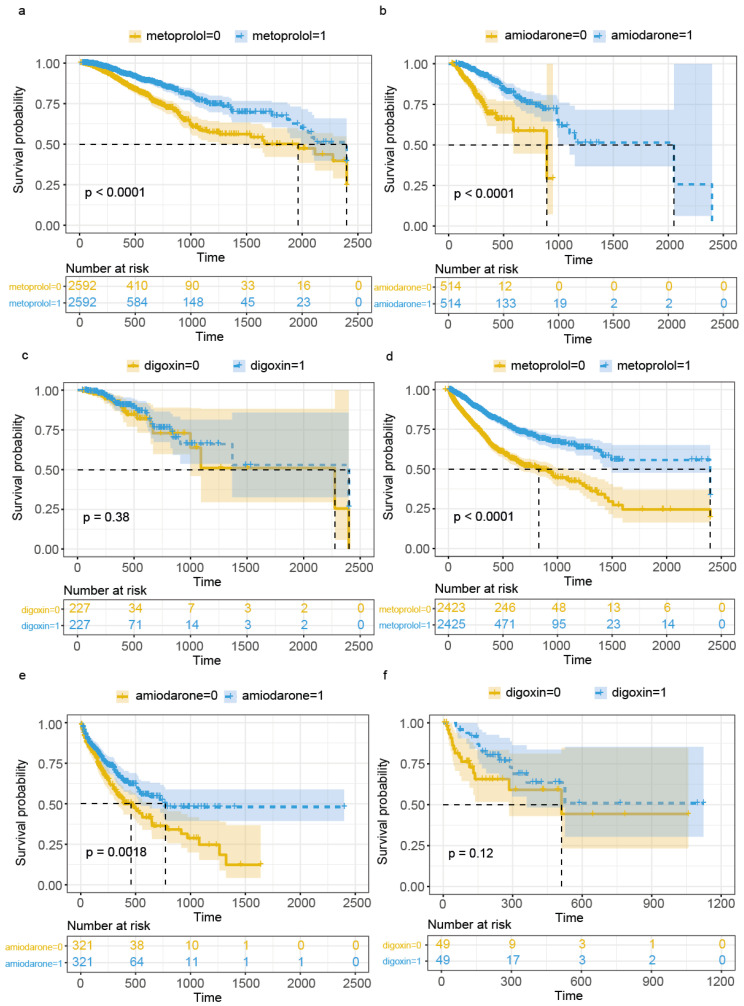
Kaplan–Meier survival curves for in-hospital mortality in AKI patients treated with antiarrhythmic medications. The patients were separately matched using propensity score matching (PSM). The x-axis represents time in hours, and the y-axis represents survival probability. The yellow lines represent patients who did not receive arrhythmia-related medications, and the blue lines represent those who received them. Shaded areas indicate 95% confidence intervals. The *p*-values from log-rank tests indicate the statistical significance of the differences between the groups. (**a**–**c**) Analysis of patients from the MIMIC-IV database: (**a**) metoprolol, (**b**) amiodarone, (**c**) digoxin. (**d**–**f**) External validation using patients from the eICU database: (**d**) metoprolol, (**e**) amiodarone, (**f**) digoxin. The number of patients at risk at different time points is shown below each graph.

We conducted a post hoc analysis to evaluate the effects of three commonly used antiarrhythmic medications (amiodarone, metoprolol, and digoxin) on outcomes in AKI patients with arrhythmia. Our primary focus was on assessing their impact on in-hospital mortality risk. To ensure a balanced comparison, we employed 1:1 propensity score matching (PSM) for each medication group before performing log-rank tests. The propensity-score-matched data are presented in Supplementary Tables S8–S10. Our analysis revealed that both amiodarone (HR:0.28 (95% CI 0.19–0.41; *p* < 0.01)) and metoprolol (HR:0.5 (95% CI 0.41–0.61; *p* < 0.01) had statistically significant effects on in-hospital mortality for AKI patients. However, digoxin showed no statistically significant impact (HR: 0.77, 95% CI 0.44–1.35; *p* = 0.38). The Kaplan–Meier survival curves for each medication group (amiodarone, metoprolol, and digoxin) are presented in Figure 5. These findings were validated using the eICU database, which produced consistent results: metoprolol (HR 0.44, 95% CI 0.38–0.5; *p* < 0.01), amiodarone (HR 0.66, 95% CI 0.51–0.86; *p* < 0.01), and digoxin (HR 0.72, 95% CI 0.34–1.53; *p* = 0.12).

To further account for the time-varying nature of drug exposure and dynamic clinical variables, we subsequently conducted Cox regression models with time-dependent covariates (Supplementary Table S11). In the time-varying Cox regression model, the use of amiodarone was significantly associated with reduced in-hospital mortality. Specifically, amiodarone exposure yielded a hazard ratio (HR) of 0.50 (95% CI: 0.30–0.83), indicating a 50% reduction in the hazard of death compared to non-exposure. This protective effect remained significant after adjusting for time-varying covariates. In contrast, metoprolol and digoxin were not significantly associated with in-hospital mortality.

Given the statistically significant association identified in the time-varying Cox model, amiodarone was considered a drug with strong evidence of a protective effect. As a sensitivity analysis, we applied a Fine–Gray competing risk model to assess the robustness of the observed association between amiodarone use and in-hospital mortality, considering discharge alive as a competing event. This analysis was prompted by the significant findings in the time-varying Cox model, which identified amiodarone as a strong candidate drug. The subdistribution hazard ratio (sHR) was 0.525 (95% CI: 0.314–0.876, *p* < 0.05), indicating that the protective association of amiodarone persisted even under the competing risk framework.

## 4. Discussion

This study provides a comprehensive analysis of arrhythmia incidence, predictive factors, and the impact of antiarrhythmic drug interventions on in-hospital mortality in critically ill patients with acute kidney injury (AKI). Utilizing large-scale, real-world data from the MIMIC-IV and eICU databases, our findings underscore the significant burden of arrhythmias in this patient population and highlight the potential benefits of targeted pharmacological interventions. The development and validation of predictive models, alongside a rigorous evaluation of antiarrhythmic drug efficacy, contribute insights for clinical practice and future research.

Our analysis confirms a high incidence of arrhythmias (40%) among critically ill AKI patients, consistent with previous literature indicating a strong association between renal dysfunction and cardiovascular complications [22,23,24,25]. The results highlight the unique nature of AKI-induced arrhythmias, aligning with previous research suggesting that life-threatening ventricular arrhythmias are rare among AKI patients [3]. An important point to note is that the key influencing factors in our prediction of whether patients develop arrhythmias primarily include the presence of concomitant heart disease, heart rate variations, and coagulation test results. We did not find a strong connection with previously hypothesized physiological factors, such as electrolyte imbalances like hyperkalemia [3]. This may be due to the fact that arrhythmias may have a genetic component, and no existing medical model currently predicts arrhythmias with high accuracy [26,27]. Abnormal clotting can increase the risk of thromboembolic events, such as heart attacks, all of which are linked to arrhythmias, especially atrial fibrillation [28].

Furthermore, our investigation into the use of antiarrhythmic medications (amiodarone) showed a statistically significant effect on patient survival outcomes. Existing research suggests that pre-admission treatment with amiodarone is associated with a reduced risk of AKI [29]. To ensure the robustness of our results, we conducted multiple sensitivity analyses. These included comparisons across different missing data imputation methods (e.g., predictive mean matching and random forest imputation), and alternative modeling approaches, such as Kaplan–Meier, time-varying Cox models, and Fine–Gray competing risk models. Notably, the protective association of amiodarone remained consistent across these analytic strategies, reinforcing its potential clinical benefit. Our research findings are consistent with previous studies, and we provide additional support through large-scale data and multi-center validation. Moreover, recent studies have shown that SGLT2 inhibitors can improve the prognosis of patients with heart failure and exert significant antiarrhythmic effects, which aligns well with the key variables identified in our study [30].

Our study’s limitations warrant consideration. While we utilized comprehensive public databases, missing data remain an inherent challenge. We addressed this using Multiple Imputation by Chained Equations (MICEs) to minimize potential bias. Additionally, despite employing propensity score matching to align patient groups, residual differences in baseline conditions cannot be entirely eliminated. The presence of noise in our data may have also impacted the accuracy of arrhythmia prediction. The sample size for malignant arrhythmias (e.g., VT/VF) was limited, precluding meaningful analysis. Future studies are needed to clarify their prognostic impact. Finally, our study did not account for the duration of antiarrhythmic drug exposure, which limits our ability to evaluate the potential impact of long-term use on long-term outcomes. As such, we cannot exclude the possibility that the duration of therapy may introduce bias in estimating the true effect of the intervention.

In conclusion, the predictive models demonstrated robust discriminatory ability for identifying arrhythmia occurrence in critically ill AKI patients, with the identified risk factors showing strong clinical relevance. Arrhythmia occurrence was significantly associated with increased risk of in-hospital mortality. Furthermore, amiodarone therapy effectively reduced the risk of in-hospital mortality in these patients.

## Figures and Tables

**Table 1:** Baseline differences between cardiac dysrhythmias and non-cardiac dysrhythmias group in MIMIC-IV.

	Cardiac Dysrhythmias (5614)	Non-Cardiac Dysrhythmias (8421)	*p*	SMD
**demographic information**				
in-hospital death (%)	636 (11.3)	587 (7.0)	<0.01	0.105
age (years)	72 [60–82]	66 [55–77]	<0.01	0.279
LOS (days (median [IQR]))	2.64 [1.43–5.01]	2.23 [1.26–4.23]	<0.01	0.166
gender, male (%)	3355 (59.8)	4871 (57.8)	0.025	0.04
**comorbidities**				
cerebral infarction (%)	500 (8.9)	699 (8.3)	0.22	0.021
CKD (%)	1980 (35.3)	2526 (30.0)	<0.01	0.119
diabetes (%)	1132 (20.2)	1833 (21.8)	0.02	0.039
heart failure (%)	2772 (49.4)	2541 (30.2)	<0.01	0.4
hypertension (%)	2361 (42.1)	3885 (46.1)	<0.01	0.081
Infarct circulation (%)	898 (16.0)	1239 (14.7)	0.041	0.036
pancreatitis (%)	176 (3.1)	265 (3.15)	1	0.003
**medication usage**				
antibiotic usage (%)	4062 (72.4)	5878 (69.8)	<0.01	0.06
blood products (%)	1958 (34.9)	2771 (32.9)	0.016	0.04
colloids (%)	1131 (20.1)	1638 (19.5)	0.321	0.014
crystalloids (%)	2998 (53.4)	4541 (53.9)	0.554	0.01
furosemide (%)	3455 (61.5)	4398 (52.2)	<0.01	0.188
insulin (%)	2283 (40.7)	3283 (39)	0.048	0.034
nitroglycerin (%)	1283 (22.9)	1671 (19.8)	<0.01	0.08
pressor (%)	1846 (32.9)	2205 (26.2)	<0.01	0.147
anticoagulant (%)	4279 (76.2)	6200 (73.6)	<0.01	0.06
sodiumbicarbonate (%)	712 (12.7)	1120 (13.3)	0.299	0.02
**laboratory examinations**				
WBC_valuenum (K/mcL (median [IQR]))	10.8 [7.6–15.3]	10.8 [7.6–15.1]	0.659	0.02
WBC_max (K/mcL (median [IQR]))	11.8 [8.2–16.7]	11.9 [8.3–16.6]	0.895	0.015
WBC_min (K/mcL (median [IQR]))	9.8 [7–13.6]	9.8 [6.9–13.4]	0.235	0.025
AG_valuenum (mEq/L (median [IQR]))	15 [12–17]	14 [12–17]	<0.01	0.053
AG_max (mEq/L (median [IQR]))	16 [13,19]	16 [13,19]	0.012	0.03
AG_min (mEq/L (median [IQR]))	14 [12–16]	13 [11–16]	<0.01	0.07
BUN_valuenum (mg/dL (median [IQR]))	30 [20–47]	27 [18–43]	<0.01	0.119
BUN_max (mg/dL (median [IQR]))	32 [21–49]	28 [19–45]	<0.01	0.111
BUN_min (mg/dL (median [IQR]))	28 [19–44]	25 [17–40]	<0.01	0.134
chloride_valuenum (mEq/L (median [IQR]))	104 [100–108]	105 [101–108]	<0.01	0.09
chloride_max (mEq/L (median [IQR]))	105 [101–109]	106 [102–110]	<0.01	0.103
chloride_min (mEq/L (median [IQR]))	103 [98–106]	103 [99–107]	<0.01	0.09
creatinine_valuenum (mg/dL (median [IQR]))	1.4 [1–2]	1.3 [1–2]	<0.01	0.04
creatinine_max (mg/dL (median [IQR]))	1.5 [1.1–2.2]	1.4 [1.1–2.2]	0.06	0.051
creatinine_min (mg/dL (median [IQR]))	1.2 [0.9–1.8]	1.3 [1.0–1.9]	<0.01	0.027
glucose_valuenum (mg/dL (median [IQR]))	126.5 [104–163]	129 [104–165]	0.02	0.055
glucose_max (mg/dL (median [IQR]))	150 [117–196]	151 [118–204]	<0.01	0.073
glucose_min (mg/dL (median [IQR]))	150 [117–196]	151 [118–204]	0.172	0.15
hemoglobin_valuenum (g/dL (median [IQR]))	10 [8.6–11.6]	10 [8.6–11.5]	0.497	0.015
hemoglobin_max (g/dL (median [IQR]))	10.5 [9.2–12]	10.5 [9.2–12]	0.732	0.01
hemoglobin_min (g/dL (median [IQR]))	9.6 [8.2–11.2]	9.6 [8.1–11.2]	0.503	0.02
INR_valuenum (median [IQR])	1.4 [1.2–1.8]	1.3 [1.1–1.6]	<0.01	0.219
INR_max (median [IQR])	1.4 [1.2–1.9]	1.3 [1.2–1.7]	<0.01	0.21
INR_min (median [IQR])	1.3 [1.2–1.6]	1.2 [1.1–1.5]	<0.01	0.206
pH_valuenum (units (median [IQR]))	7.37 [7.32–7.42]	7.37 [7.32–7.42]	<0.01	0.026
pH_max (units (median [IQR]))	7.4 [7.36–7.44]	7.4 [7.35–7.44]	<0.01	0.045
pH_min (units (median [IQR]))	7.35 [7.29–7.4]	7.35 [7.28–7.4]	0.09	0.022
PLT_valuenum (K/uL (median [IQR]))	182 [129–247]	186 [128–255]	<0.01	0.034
PLT_max (K/uL (median [IQR]))	190 [138–255]	192 [137–261]	<0.01	0.029
PLT_min (K/uL (median [IQR]))	168 [116–232]	173 [116–237]	0.104	0.029
potassium_valuenum (mEq/L (median [IQR]))	4.2 [3.8–4.8]	4.2 [3.8–4.7]	0.024	0.035
potassium_max (mEq/L (median [IQR]))	4.5 [4–5]	4.4 [4–5]	0.021	0.035
potassium_min (mEq/L (median [IQR]))	3.9 [3.5–4.3]	3.9 [3.5–4.3]	0.074	0.029
PT_valuenum (sec (median [IQR]))	15.3 [13.2–19.4]	14.3 [12.6–16.9]	<0.01	0.254
PT_max (sec (median [IQR]))	15.7 [13.4–20.1]	14.5 [12.7–17.6]	<0.01	0.23
PT_min (sec (median [IQR]))	14.5 [12.8–18]	13.7 [12.3–16.1]	<0.01	
sodium_valuenum (mEq/L (median [IQR]))	138 [135–141]	138 [135–141]	0.384	0.018
sodium_max (mEq/L (median [IQR]))	139 [137–142]	140 [137–142]	0.139	0.019
sodium_min (mEq/L (median [IQR]))	137 [134–140]	137 [134–140]	0.41	0.019
bicarbonate_valuenum (mEq/L (median [IQR]))	22 [19–25]	22 [19–25]	<0.01	0.08
bicarbonate_max (mEq/L (median [IQR]))	23 [21–26]	23 [21–26]	<0.01	0.09
bicarbonate_min (mEq/L (median [IQR]))	23 [21–26]	23 [21–26]	<0.01	0.085
totalcalcium_valuenum (mg/dL (median [IQR]))	8.3 [7.8–8.8]	8.3 [7.8–8.8]	<0.01	0.046
totalcalcium_max (mg/dL (median [IQR]))	8.5 [8–8.9]	8.4 [8–8.9]	0.07	0.022
totalcalcium_min (mg/dL (median [IQR]))	8.2 [7.6–8.7]	8.1 [7.6–8.6]	<0.01	0.059
RBC_valuenum (m/uL (median [IQR]))	3.39 [2.9–3.96]	3.39 [2.89–3.93]	0.30	0.025
RBC_max (m/uL (median [IQR]))	3.53 [3.09–4.04]	3.51 [3.07–4.01]	0.163	0.032
RBC_min (m/uL (median [IQR]))	3.26 [2.77–3.8]	3.23 [2.76–3.77]	0.06	0.041
**vital signs**				
APSIII (median [IQR])	51 [40–67]	47 [37–62]	<0.01	0.154
HR_valuenum (bpm/min (median [IQR]))	90 [76–108]	86 [76–99]	<0.01	0.188
HR_max (bpm/min (median [IQR]))	107 [89–123]	99 [88–111]	<0.01	0.273
HR_min (bpm/min (median [IQR]))	72 [60–86]	70 [61–79]	<0.01	0.203
MAP_valuenum (mmHg (median [IQR]))	80 [69–93]	81 [70–94]	<0.01	0.064
MAP_max (mmHg (median [IQR]))	100 [89–114]	101 [90–115]	<0.01	0.036
MAP_min (mmHg (median [IQR]))	57 [50–65]	58 [51–66]	<0.01	0.100
RR_valuenum (insp/min (median [IQR]))	19 [16–24]	19 [15–23]	<0.01	0.08
RR_max (insp/min (median [IQR]))	28 [24–32]	27 [24–31]	<0.01	0.142
RR_min (insp/min (median [IQR]))	13 [10–15]	12 [10–15]	<0.01	0.063
temperature_valuenum (°F (median [IQR]))	98 [97.4–98.6]	98.1 [97.5–98.7]	<0.01	0.046
temperature_max (°F (median [IQR]))	98.8 [98.3–99.6]	98.9 [98.4–99.7]	<0.01	0.047
temperature_min (°F (median [IQR]))	97.5 [96.7–97.9]	97.6 [96.9–98]	<0.01	0.055
output_valuenum (ml/min (median [IQR]))	145 [60–275]	150 [65–300]	<0.01	0.087
output_max (ml/min (median [IQR]))	275 [150–400]	300 [160–450]	<0.01	0.08
output_min (ml/min (median [IQR]))	30 [15–60]	30 [15–75]	<0.01	0.085
**treatment measures**				
CRRT (%)	411 (7.3)	598 (7.1)	0.645	0.008
ventilation (%)	3712 (66.1)	5042 (59.9)	<0.01	0.13

_valuenum is the initial value of patients, __min was calculated based on minimum values obtained during the first 24 h in the ICU, and_max was calculated based on maximum values obtained during the ICU stay. LOS length of stay, CKD chronic kidney disease, WBC white blood cells, AG anion gap, BUN blood urea nitrogen, INR international normalized ratio, PLT platelet, PT prothrombin time, RBC red blood cells, APSIII acute physiology score III, MAP mean arterial pressure, HR heart rate, RR respiratory rate, CRRT renal replacement therapy, IQR interquartile range, SMD standardized mean difference.

**Table 2:** XGBoost model validation results.

	Accuracy	Precision	Recall	F1	Brier
**Overall**					
Train	0.900	0.871	0.880	0.876	0.103
Internal validation	0.671	0.640	0.616	0.597	0.203
External validation	0.616	0.632	0.577	0.586	0.217
**Atrial Fibrillation**					
Train	0.85	0.695	0.685	0.555	0.083
Internal validation	0.81	0.525	0.552	0.434	0.095
External validation	0.79	0.443	0.454	0.378	0.109
**Sinus bradycardia**					
Train	0.92	0.480	0.606	0.343	0.0416
Internal validation	0.91	0.420	0.551	0.305	0.0422
External validation	0.90	0.300	0.504	0.230	0.0358
**Sinus Tachycardia**					
Train	0.89	0.613	0.377	0.467	0.079
Internal validation	0.89	0.561	0.359	0.438	0.082
External validation	0.88	0.383	0.360	0.370	0.056

Bold formatting was used to indicate separate modules.

**Table 3:** Analysis of the association between different types of arrhythmias and in-hospital mortality (univariate analysis).

	Atrial Fibrillation	Sinus Tachycardia	Sinus Bradycardia	AV Block	Atrial Flutter
Before PSM	<0.01	<0.01	<0.01	0.165	0.176
After PSM	<0.01	<0.01	0.755	0.309	0.497

AV Block: atrioventricular (AV) block.

## Data Availability

The datasets analyzed during the current study are publicly available in the MIMIC IV and the eICU databases. All data generated during this study are included in this published article.

## Supplementary Materials

The following supporting information can be downloaded at: https://www.mdpi.com/article/10.3390/jcm14134552/s1, Figure S1: Bee swarm plot illustrating the SHAP values for various features in the model; Figure S2: Detailed SHAP value distribution for each feature within the XGBoost model; Figure S3: Feature importance derived from the Atrial Fibrillation XGBoost model; Figure S4: Feature importance derived from the sinus bradycardia XGBoost model; Figure S5: Feature importance derived from the Sinus Tachycardia XGBoost model; Figure S6: Calibration curves of the BIC predicted model in (a) the MIMIC-IV and (b) the eICU validation set; Table S1: missing data summary; Table S2: convergence table; Table S3: atrial fibrillation PSM table; Table S4: atrial flutter PSM table; Table S5: AV block PSM table; Table S6: sinus tachycardia PSM table; Table S7: sinus bradycardia PSM table; Table S8: amiodarone PSM data; Table S9: digoxin PSM data; Table S10: metoprolol PSM data; Table S11: cox time varying results table.

## References

[1] Uchino S., Kellum J.A., Bellomo R., Doig G.S., Morimatsu H., Morgera S., Schetz M., Tan I., Bouman C., Macedo E. (2005). Acute Renal Failure in Critically Ill Patients: A Multinational, Multicenter Study. JAMA.

[2] Patschan D., Marahrens B., Jansch M., Patschan S., Ritter O. (2022). Experimental Cardiorenal Syndrome Type 3: What Is Known so Far?. J. Clin. Med. Res..

[3] Genovesi S., Regolisti G., Burlacu A., Covic A., Combe C., Mitra S., Basile C., EuDial Working Group of ERA (2023). The Conundrum of the Complex Relationship between Acute Kidney Injury and Cardiac Arrhythmias. Nephrol. Dial. Transplant..

[4] Faubel S., Shah P.B. (2016). Immediate Consequences of Acute Kidney Injury: The Impact of Traditional and Nontraditional Complications on Mortality in Acute Kidney Injury. Adv. Chronic Kidney Dis..

[5] Heinz G. (2008). Arrhythmias in the ICU. Am. J. Respir. Crit. Care Med..

[6] Shawwa K., Kompotiatis P., Bobart S.A., Mara K.C., Wiley B.M., Jentzer J.C., Kashani K.B. (2021). New-Onset Atrial Fibrillation in Patients with Acute Kidney Injury on Continuous Renal Replacement Therapy. J. Crit. Care.

[7] Hellman T., Uusalo P., Järvisalo M.J. (2022). New-Onset Atrial Fibrillation in Critically Ill Acute Kidney Injury Patients on Renal Replacement Therapy. Europace.

[8] Singbartl K., Kellum J.A. (2012). AKI in the ICU: Definition, Epidemiology, Risk Stratification, and Outcomes. Kidney Int..

[9] Zhou J., Yang L., Zhang K., Liu Y., Fu P. (2012). Risk Factors for the Prognosis of Acute Kidney Injury under the Acute Kidney Injury Network Definition: A Retrospective, Multicenter Study in Critically Ill Patients. Nephrology.

[10] Lee S.A., Cozzi M., Bush E.L., Rabb H. (2018). Distant Organ Dysfunction in Acute Kidney Injury: A Review. Am. J. Kidney Dis..

[11] Basu R.K., Wheeler D.S. (2013). Kidney-Lung Cross-Talk and Acute Kidney Injury. Pediatr. Nephrol..

[12] Angeli P., Garcia-Tsao G., Nadim M.K., Parikh C.R. (2019). News in Pathophysiology, Definition and Classification of Hepatorenal Syndrome: A Step beyond the International Club of Ascites (ICA) Consensus Document. J. Hepatol..

[13] Johnson A.E.W., Bulgarelli L., Shen L., Gayles A., Shammout A., Horng S., Pollard T.J., Hao S., Moody B., Gow B. (2023). MIMIC-IV, a Freely Accessible Electronic Health Record Dataset. Sci. Data.

[14] Pollard T.J., Johnson A.E.W., Raffa J.D., Celi L.A., Mark R.G., Badawi O. (2018). The eICU Collaborative Research Database, a Freely Available Multi-Center Database for Critical Care Research. Sci. Data.

[15] Lee K.J., Carlin J.B. (2010). Multiple Imputation for Missing Data: Fully Conditional Specification versus Multivariate Normal Imputation. Am. J. Epidemiol..

[16] Zhang Z. (2016). Missing Data Imputation: Focusing on Single Imputation. Ann. Transl. Med..

[17] Zhang Z. (2016). Univariate Description and Bivariate Statistical Inference: The First Step Delving into Data. Ann. Transl. Med..

[18] Zhang Z. (2016). Variable Selection with Stepwise and Best Subset Approaches. Ann. Transl. Med..

[19] Chen T., Guestrin C. (2016). XGBoost: A Scalable Tree Boosting System. Proceedings of the 22nd ACM SIGKDD International Conference on Knowledge Discovery and Data Mining.

[20] Lundberg S.M., Erion G., Chen H., DeGrave A., Prutkin J.M., Nair B., Katz R., Himmelfarb J., Bansal N., Lee S.-I. (2020). From Local Explanations to Global Understanding with Explainable AI for Trees. Nat. Mach. Intell..

[21] Jin Y., Kattan M.W. (2023). Methodologic Issues Specific to Prediction Model Development and Evaluation. Chest.

[22] Annane D., Sébille V., Duboc D., Le Heuzey J.-Y., Sadoul N., Bouvier E., Bellissant E. (2008). Incidence and Prognosis of Sustained Arrhythmias in Critically Ill Patients. Am. J. Respir. Crit. Care Med..

[23] Reinelt P., Karth G.D., Geppert A., Heinz G. (2001). Incidence and Type of Cardiac Arrhythmias in Critically Ill Patients: A Single Center Experience in a Medical-Cardiological ICU. Intensive Care Med..

[24] Goodman S., Shirov T., Weissman C. (2007). Supraventricular Arrhythmias in Intensive Care Unit Patients: Short and Long-Term Consequences. Anesth. Analg..

[25] Trappe H.-J., Brandts B., Weismueller P. (2003). Arrhythmias in the Intensive Care Patient. Curr. Opin. Crit. Care.

[26] Cheung C.C., Tadros R., Davies B., Krahn A.D. (2020). Genetic Testing in Inherited Arrhythmias: Approach, Limitations, and Challenges. Can. J. Cardiol..

[27] Roberts J.D., Vittinghoff E., Lu A.T., Alonso A., Wang B., Sitlani C.M., Mohammadi-Shemirani P., Fornage M., Kornej J., Brody J.A. (2021). Epigenetic Age and the Risk of Incident Atrial Fibrillation. Circulation.

[28] Nieuwlaat R., Connolly B.J., Hubers L.M., Cuddy S.M., Eikelboom J.W., Yusuf S., Connolly S.J. (2012). Quality of Individual INR Control and the Risk of Stroke and Bleeding Events in Atrial Fibrillation Patients: A Nested Case Control Analysis of the ACTIVE W Study. Thromb. Res..

[29] Gill J., Heel R.C., Fitton A. (1992). Amiodarone. An Overview of Its Pharmacological Properties, and Review of Its Therapeutic Use in Cardiac Arrhythmias. Drugs.

[30] Mariani M.V., Lavalle C., Palombi M., Pierucci N., Trivigno S., D’Amato A., Filomena D., Cipollone P., Laviola D., Piro A. (2025). SGLT2i Reduce Arrhythmic Events in Heart Failure Patients with Cardiac Implantable Electronic Devices. ESC Heart Fail..

