# Supplementary material for: Evaluating In-Hospital Arrhythmias in Critically Ill Acute Kidney Injury Patients: Predictive Models, Mortality Risks, and the Efficacy of Antiarrhythmic Drugs
Source: J Clin Med. 2025 Jun 26;14(13):4552. doi: 10.3390/jcm14134552 (PMC12249616; doi:10.3390/jcm14134552)
Supplement: Supplementary file 1 [file jcm-14-04552-s001.zip › Supplementary Data.pdf]

SUPPLEMENTARY DATA

Evaluating in-hospital arrhythmias in critically ill acute kidney injury patients: predictive models, mortality risks, and the efficacy of antiarrhythmic drugs

Wanqiu Xie, MSc [1], Henriette Franz, PhD [2], Toma Antonov Yakulov, PhD [1,*]

[1] Renal Division, University Freiburg Medical Center, Faculty of Medicine, University of Freiburg, Germany

[2] Department of Biomedicine, University of Basel, Pestalozzistr. 20, Basel CH-4056, Switzerland

* To whom correspondence should be addressed:
Toma Antonov Yakulov
Renal Division
University Freiburg Medical Center – IMITATE
79106 Freiburg, Germany
E-mail: toma.antonov.yakulov@uniklinik-freiburg.de
Tel: +49 761 270 63036

R packages were used in this study

Data manipulation and cleaning

- **moments**: For calculating statistical moments.
- **mice**: For multiple imputation of missing data.
- **reshape2**: For reshaping data between wide and long formats.

Statistical analysis and modeling

- **caret**: For training and tuning machine learning models.
- **glmnet**: For fitting generalized linear models via penalized maximum likelihood.
- **leaps**: For regression subset selection.
- **car**: For companion functions to applied regression.
- **MASS**: For functions and datasets to support Venables and Ripley's MASS.
- **rms**: For regression modeling strategies.
- **rmda**: For risk model decision analysis (DCA).
- **MatchIt**: For matching treatment and control groups in observational studies.
- **survival**: For survival analysis, Time-varying covariate
- **survminer**: For visualizing survival analysis results.
- Cmprsk: Fine-Gray model

Visualization

- **corrplot**: For visualizing correlation matrices.
- **ggplot2**: For creating elegant data visualizations.
- **shapviz**: For visualizing SHAP (SHapley Additive exPlanations) values.

Machine learning and model evaluation

- **InformationValue**: For model performance evaluation.
- **pROC**: For visualizing and analyzing ROC curves.
- **randomForest**: For implementing random forest algorithms.
- **xgboost**: For extreme gradient boosting.
- **ROCR**: For visualizing the performance of scoring classifiers.

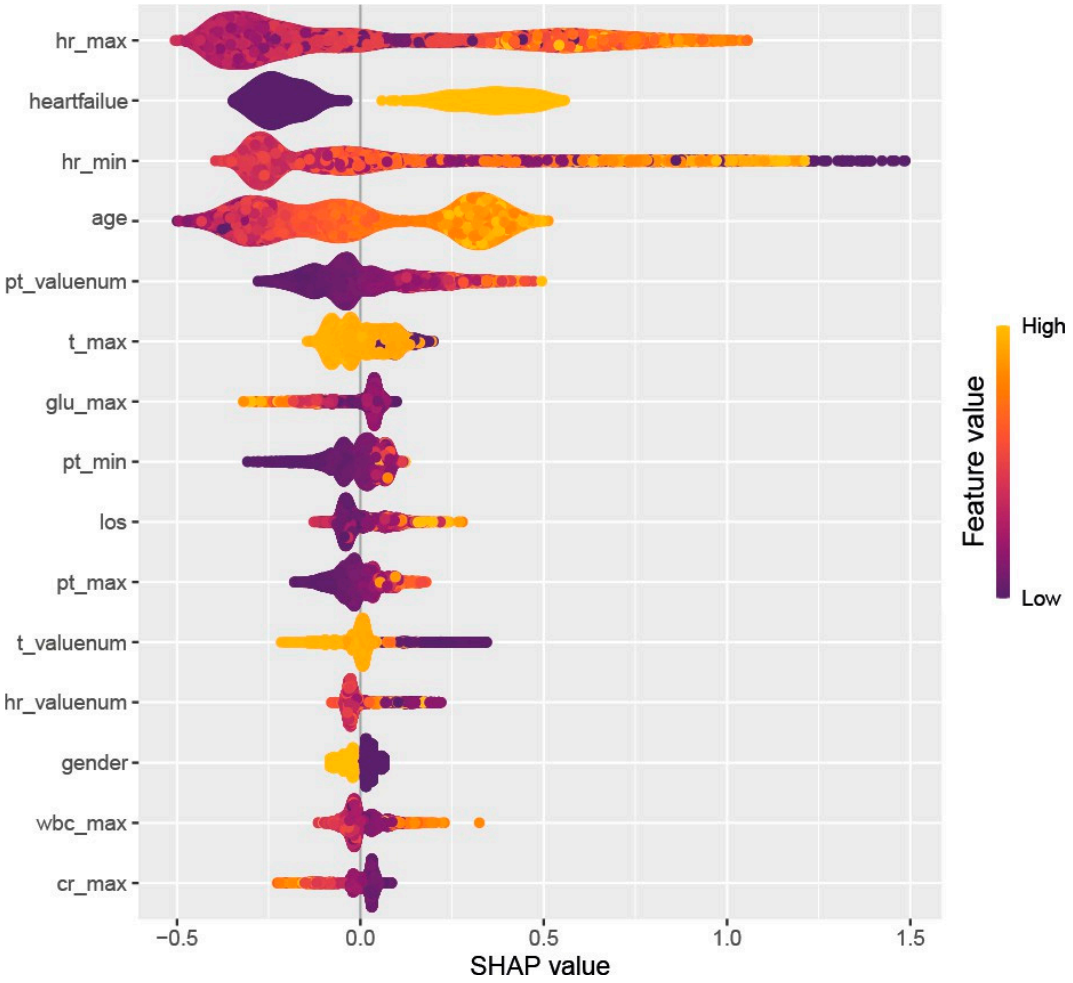

Fig. S1 Bee swarm plot illustrating the SHAP values for various features in the model. Each point in the plot represents a sample, with the SHAP value on the horizontal axis and the feature name on the vertical axis. The SHAP value indicates the impact of each feature on the model's output, with higher values suggesting a greater influence. The density of the points along the horizontal axis shows the distribution of SHAP values for each feature. Key variables are abbreviated as follows: hr = heart rate, map = mean arterial pressure, plt = platelet, glu = glucose, t = temperature, pt = prothrombin time, ckd = chronic kidney disease,wbc=white blood cell, cr=creatinine. Suffixes: _valuenum indicates the initial value, _min represents the minimum value, and _max represents the maximum value obtained during the first 24 hours in the ICU.

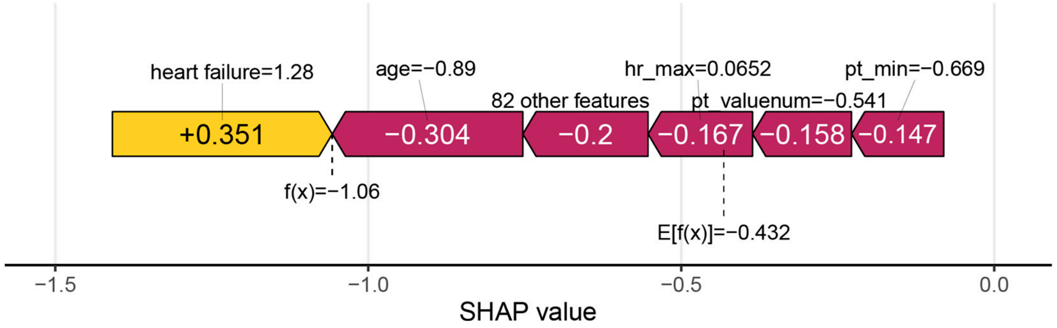

Fig. S2 Detailed SHAP value distribution for each feature within the XGBoost model. The SHAP values indicate the direction and magnitude of each feature's impact on the model's predictions. Red bars represent a decrease, and yellow bars represent an increase in the risk of arrhythmia. The function f(x) represents the model's predicted outcome based on the input variables x, while E[f(x)] denotes the expected value of the model's predictions. Abbreviations: hr = heart rate, pt = prothrombin time.Suffixes: _valuenum indicates the initial value, _min represents the minimum value, and _max represents the maximum value obtained during the first 24 hours in the ICU.

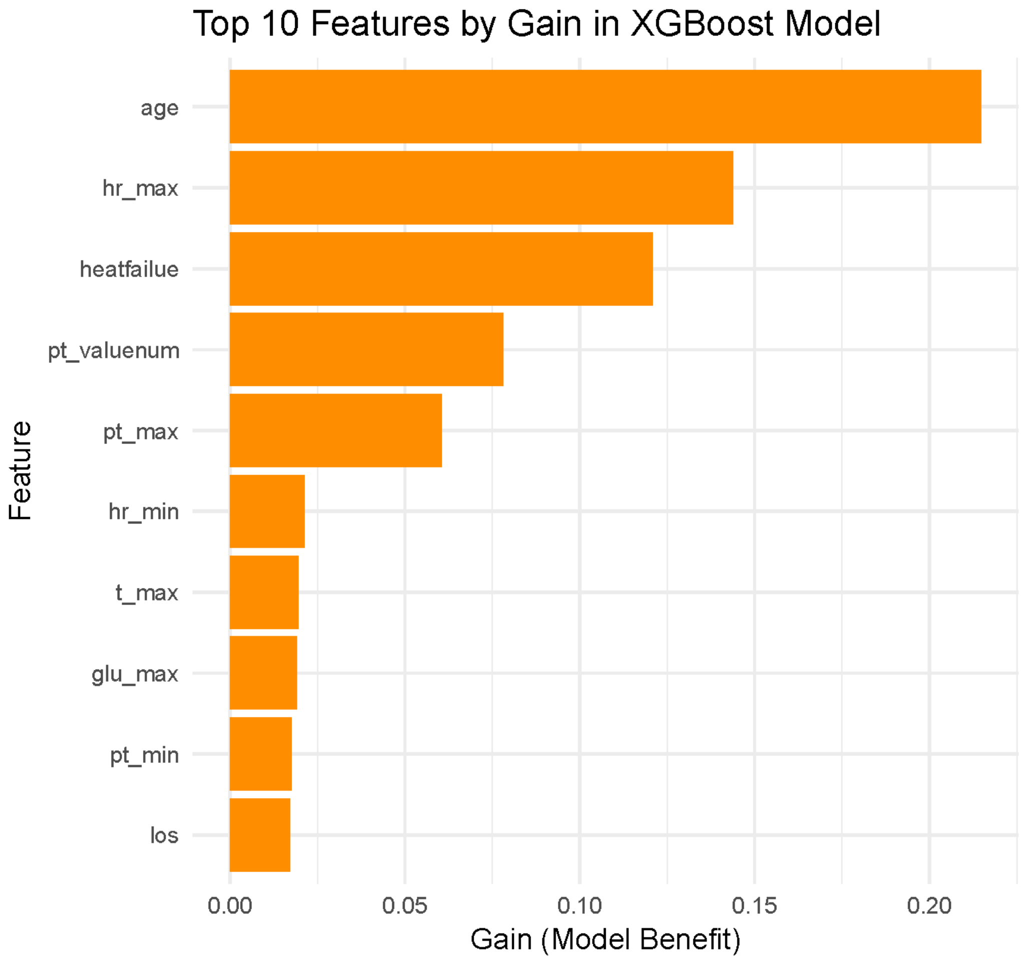

Fig. S3 Feature importance derived from the Atrial Fibrillation XGBoost model. The x-axis represents the gain, which indi-cates the relative contribution of each feature to the model. Key features include hr_max, heart-failure,hr_min, age,and pt_valuenum. Abbreviations: hr = heart rate, map = mean arterial pressure, plt = platelet, glu = glucose, t = temperature, pt = prothrombin time, ckd = chronic kidney dis-ease,wbc=white blood cell, cr=creatinine. Suffixes: _valuenum indicates the initial value, _min represents the minimum value, and _max represents the maximum value obtained during the first 24 hours in the ICU.

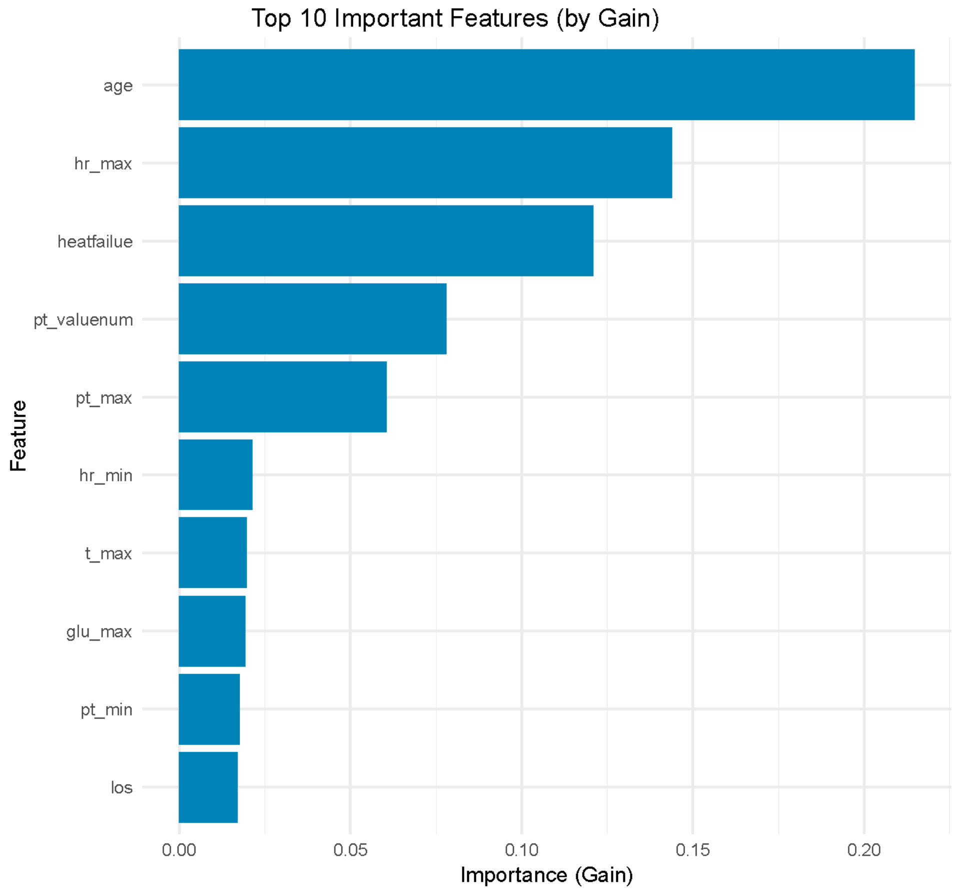

Fig. S4 Feature importance derived from the sinus bradycardia XGBoost model. The x-axis represents the gain, which indi-cates the relative contribution of each feature to the model. Key features include hr_max, heart-failure,hr_min, age,and pt_valuenum. Abbreviations: hr = heart rate, map = mean arterial pressure, plt = platelet, glu = glucose, t = temperature, pt = prothrombin time, ckd = chronic kidney dis-ease,wbc=white blood cell, cr=creatinine. Suffixes: _valuenum indicates the initial value, _min represents the minimum value, and _max represents the maximum value obtained during the first 24 hours in the ICU.

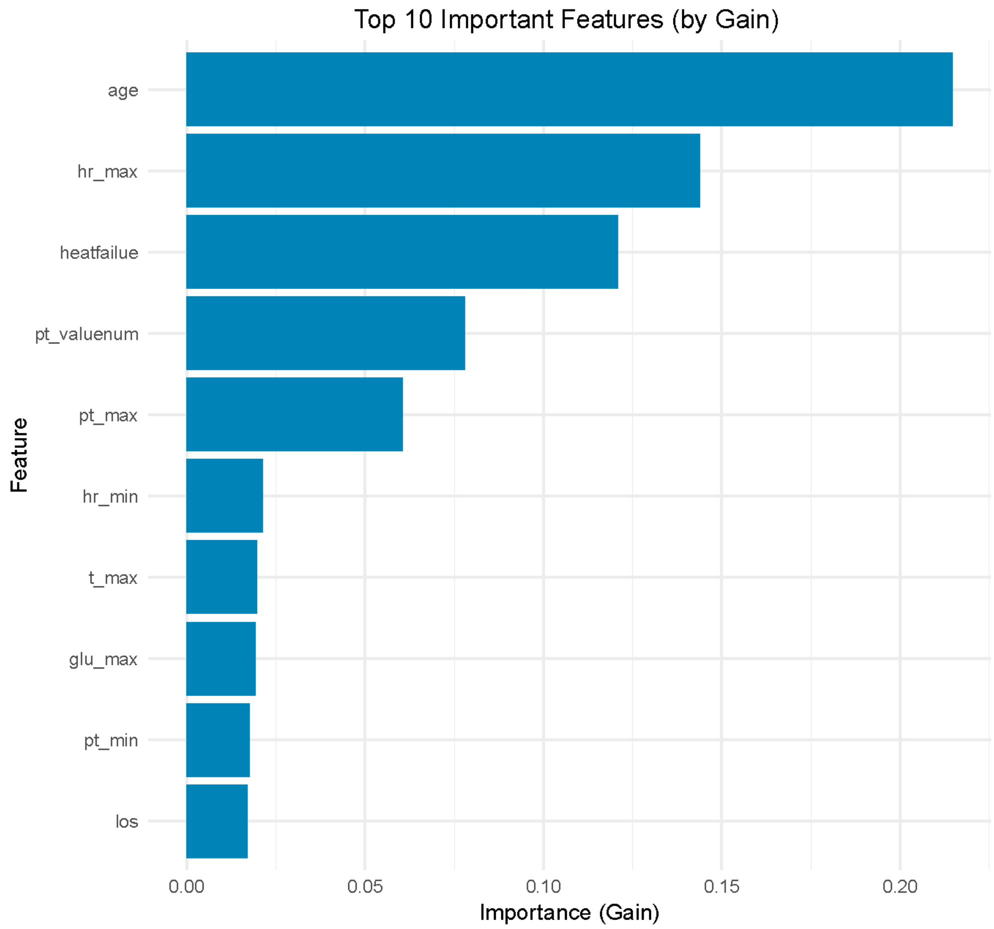

Fig. S5 Feature importance derived from the Sinus Tachycardia XGBoost model. The x-axis represents the gain, which indi-cates the relative contribution of each feature to the model. Key features include hr_max, heart-failure,hr_min, age,and pt_valuenum. Abbreviations: hr = heart rate, map = mean arterial pressure, plt = platelet, glu = glucose, t = temperature, pt = prothrombin time, ckd = chronic kidney dis-ease,wbc=white blood cell, cr=creatinine. Suffixes: _valuenum indicates the initial value, _min represents the minimum value, and _max represents the maximum value obtained during the first 24 hours in the ICU.

a

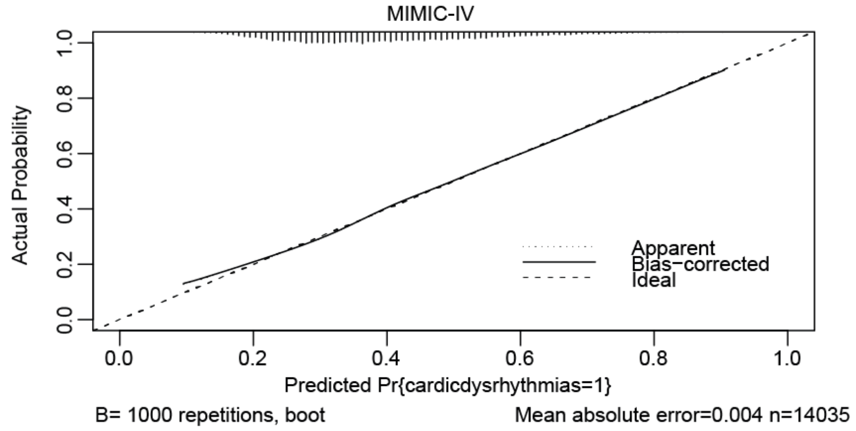

b

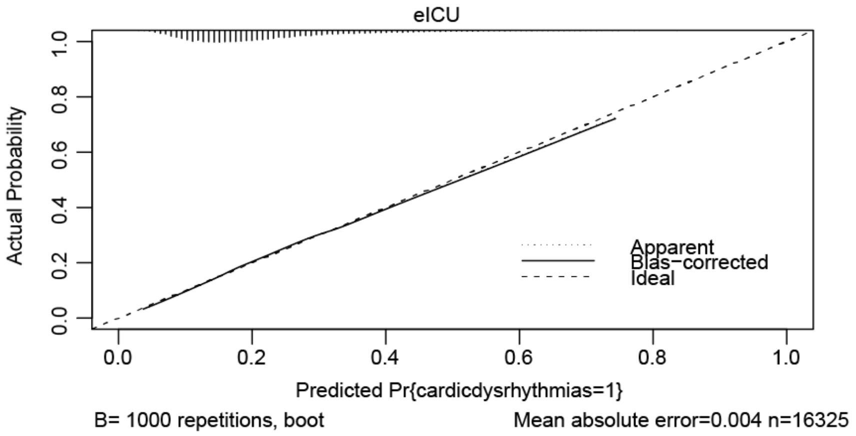

Fig. S6. Calibration curves of the BIC predicted model in (a) the MIMIC-IV and (b) the eICU validation set. The x-axis represents the predicted probability of in-hospital arrhythmias calculated by the BIC model, while the y-axis shows the observed actual probability. The solid curve represents the apparent performance in the initial cohort, and the dotted curve is bias-corrected by bootstrapping (B = 1000 repetitions), demonstrating the predicted model's performance. The diagonal dashed line indicates perfect calibration.
