# Supplementary material for: Evaluating In-Hospital Arrhythmias in Critically Ill Acute Kidney Injury Patients: Predictive Models, Mortality Risks, and the Efficacy of Antiarrhythmic Drugs
Source: J Clin Med. 2025 Jun 26;14(13):4552. doi: 10.3390/jcm14134552 (PMC12249616; doi:10.3390/jcm14134552)
Supplement: Supplementary file 1 [file jcm-14-04552-s001.zip › Supplementary Table S1.pdf]

Missing Data Percentage Table

Variable	MIMIC Missing (%)	eICU Missing (%)
los	0.0	0.0
age	0.0	0.0
In-hospital death	0.0	0.0
gender	0.0	0.0
ag_valuenum	1.6031	1.2
ag_max	1.6031	1.2
ag_min	1.6031	1.2
chloridevaluenum	1.4321	0.9
chloride_max	1.4321	0.9
chloride_min	1.4321	0.9
bun_valuenum	0.9334	1.1
bun_max	0.9191	1.1
bun_min	0.9191	1.1
cr_valuenum	0.8906	1.3
cr_max	0.8835	1.3
cr_min	0.8835	1.3
glu_valuenum	1.5034	0.8
glu_max	1.5034	0.8
glu_min	1.5034	0.8
hemoglobin_valuenum	2.2943	1.6
hemoglobin_max	2.2943	1.6
hemoglobin_min	2.2943	1.6
inr_valuenum	15.9601	10.3
inr_max	15.3901	10.3

inr_min	15.3901	10.3
ph_valuenum	26.2451	15.3
ph_max	26.2451	15.3
ph_min	26.2451	15.3
plt_valuenum	2.2943	1.9
plt_max	2.2943	1.9
plt_min	2.2943	1.9
potassium_valuenum	1.368	0.6
potassium_max	1.368	0.6
potassium_min	1.368	0.6
pt_valuenum	15.8247	12.1
pt_max	15.8247	12.1
pt_min	15.8247	12.1
rbc_valuenum	1.9736	0.7
rbc_max	1.6103	0.7
rbc_min	1.6103	0.7
sodium_valuenum	1.4963	0.3
sodium_max	1.4963	0.3
sodium_min	1.4963	0.3
wbc_valuenum	1.995	1.2
wbc_max	1.6031	1.2
wbc_min	1.6031	1.2
bicarbonate_valuenum	1.0046	0.8
bicarbonate_max	0.9263	0.8
bicarbonate_min	0.9263	0.8
totalcalcium_valuenum	6.0349	8.5

totalcalcium_max	6.0278	8.5
totalcalcium_min	6.0278	8.5
hr_valuenum	0.1211	0.22
hr_max	0.1211	0.22
hr_min	0.1211	0.22
map_valuenum	0.4631	0.11
map_max	0.4631	0.11
map_min	0.4631	0.11
rr_valuenum	0.1853	0.17
rr_max	0.1853	0.17
rr_min	0.1853	0.17
t_valuenum	2.7645	3.32
t_max	2.7645	3.32
t_min	2.7645	3.32
output	3.9259	4.50
output_max	3.9259	4.50
output_min	3.9259	4.50
crrt	0.0	0.0
vent	0.0	0.0
apsiii	0.0	0.0
cerebralinfarction	0.0	0.0
ckd	0.0	0.0
dm	0.0	0.0
heatfailue	0.0	0.0
hypertension	0.0	0.0
hypotension	0.0	0.0

myocardialinfarction	0.0	0.0
pancreatitis	0.0	0.0
antibiotics	0.0	0.0
bloodproducts	0.0	0.0
colloids	0.0	0.0
crystalloids	0.0	0.0
furosemide	0.0	0.0
insulin	0.0	0.0
anticoagulant	0.0	0.0
nitroglycerin	0.0	0.0
pressor	0.0	0.0
sodiumbicarbonate	0.0	0.0
